# Developmental fluoxetine exposure in zebrafish reduces offspring basal cortisol concentration via life stage-dependent maternal transmission

**DOI:** 10.1371/journal.pone.0212577

**Published:** 2019-02-21

**Authors:** Rubén Martinez, Marilyn N. Vera-Chang, Majd Haddad, Jessica Zon, Laia Navarro-Martin, Vance L. Trudeau, Jan A. Mennigen

**Affiliations:** 1 Department of Environmental Chemistry, IDAEA-CSIC, Jordi Girona, Barcelona, Spain; 2 Department of Cellular Biology, Physiology and Immunology, Universitat de Barcelona (UB), Barcelona, Spain; 3 Department of Biology, University of Ottawa, Ottawa, Ontario, Canada; University of Missouri Columbia, UNITED STATES

## Abstract

Fluoxetine (FLX) is a pharmaceutical used to treat affective disorders in humans, but as environmental contaminant also affects inadvertently exposed fish in urban watersheds. In humans and fish, acute FLX treatment and exposure are linked to endocrine disruption, including effects on the reproductive and stress axes. Using the zebrafish model, we build on the recent finding that developmental FLX exposure reduced cortisol production across generations, to determine possible parental and/or life-stage-dependent (age and/or breeding experience) contributions to this phenotype. Specifically, we combined control and developmentally FLX-exposed animals of both sexes (F_0_) into four distinct breeding groups mated at 5 and 9 months, and measured offspring (F_1_) basal cortisol at 12 dpf. Basal cortisol was lower in F_1_ descended from developmentally FLX-exposed F_0_ females bred at 5, but not 9 months, revealing a maternal, life-stage dependent effect. To investigate potential molecular contributions to this phenotype, we profiled maternally deposited transcripts involved in endocrine stress axis development and regulation, epigenetic (*de novo* DNA methyltransferases) and post-transcriptional (miRNA pathway components and specific miRNAs) regulation of gene expression in unfertilized eggs. Maternal FLX exposure resulted in decreased transcript abundance of glucocorticoid receptor, *dnmt3* paralogues and miRNA pathway components in eggs collected at 5 months, and increased transcript abundance of miRNA pathway components at 9 months. Specific miRNAs predicted to target stress axis transcripts decreased (*miR-740*) or increased (*miR-26*, *miR-30d*, *miR-92a*, *miR-103*) in eggs collected from FLX females at 5 months. Increased abundance of *miRNA-30d* and *miRNA-92a* persisted in eggs collected from FLX females at 9 months. Clustering and principal component analyses of egg transcript profiles separated eggs collected from FLX-females at 5 months from other groups, suggesting that oocyte molecular signatures, and miRNAs in particular, may serve as predictive tools for the offspring phenotype of reduced basal cortisol in response to maternal FLX exposure.

## 1. Introduction

Selective serotonin reuptake inhibitors (SSRIs) are widely prescribed pharmaceuticals used to treat mood disorders [1]. Prescriptions of SSRIs have doubled in the past decades in many countries, reaching prescription rates as high as 10–15% of the adult population, with up to 2-fold higher prescription rates in women [2–6]. This raises concerns about potential effects of perinatal SSRI exposure in the offspring [7], as in pregnant or nursing women, prescription rates of 1–10% have been reported [8,9]. In parallel with spiking prescription rates, SSRIs have been increasingly found in wastewater-effluent receiving urban streams [10], reaching total SSRI concentrations in the range of low μg/L (ppb, parts per billion) immediately downstream of point sources of waste water treatment plant (WTTP) effluents [11,12]. Because SSRIs are bioconcentrated in fish [12–15], a concern for SSRIs is the environmental exposure of inadvertently exposed aquatic wildlife [16], especially since the the serotonergic system is well conserved between fish and mammals [17,18]. This raises the possibility of SSRI-dependent effects through modulation of the serotonergic system in both vertebrate classes [10,16,19]. In fish [20], as in mammals [21], one of several roles of serotonin is the regulation of the endocrine system, including the stress axis [22,23].

As the first SSRI on the market, fluoxetine (FLX), originally marketed as Prozac [24], continues to be prescribed as generic pharmacological treatment for major depression, as well as additional conditions such as obsessive-compulsive disorder [25], anxiety [26], pre-menstrual dysphoric disorder [27], and eating disorders [28]. FLX remains the most studied SSRI with regard to both human health [29] and aquatic toxicology [30]. In human patients, FLX kinetics are well described: orally administered FLX is almost completely absorbed, but less than 90% are bioavailable because of first-pass metabolism and a high distribution volume [1,31]. FLX and its active metabolite norfluoxetine (NFLX) have a half-life of 1–4 d and 7–15 d, respectively, and exhibit non-linear kinetics [1,31]. Following a one-month administration of 40 mg FLX per day, human plasma concentrations reach approximately 100–300 μg/L FLX and 75–250 μg/L NFLX, respectively [31]. Offspring may be directly exposed during its development as fetus or infants, owing to the fact that FLX and NFLX can cross the human placenta [32] and are excreted via breast milk [33,34]. Overall, infant serum concentrations of FLX and NFLX have been reported at concentrations of 20–250 μg/L [32–34]. Animal studies corroborate these findings, revealing that FLX and NFLX have been detected in fetal brain tissue at low μg/ml concentrations in rats after single or repeated administration of 12 mg/kg FLX in pregnant dams [32].

Human excretion of up to 10% of FLX parent compound and conjugated FLX glucuronide via the urine [1] and/or improper disposal have been reported to result in untreated urban WWTP influent concentrations of FLX as high as 3 μg/L [11]. In exposed fish, bioconcentration occurs and can reach factors >100, especially in slightly alkaline water conditions [13,15,35]. Tissue concentrations of FLX and its active metabolite NFLX are highest in brain and liver of wild-caught fish, reaching levels as high as 10 ng/g for FLX and 20 ng/g for NFLX [11,12,14]. Whether FLX is transferred into eggs during female vitellogenesis and oocyte maturation before spawning is unknown, but in externally fertilizing fish, gametes and zygote can directly be exposed to FLX in the water.

In both fish [20,36–38] and mammals [39,40] endocrine disrupting effects of FLX have been reported at and below human therapeutic plasma (equivalent) concentrations, which include the endocrine stress axis function [41–46]. While developmental [47–53] and adult [16,20,46,54–56] consequences of FLX exposure have been comparatively well studied in fish at different levels of biological organization [15], intergenerational effects of developmental FLX exposure have only recently been described in zebrafish [57]. This study revealed that developmental FLX exposure has the capacity to differentially reduce basal cortisol concentrations or blunt mechanical stressor induced cortisol concentrations in subsequent generations. However, parental and life-stage (age- and/or breeding experience specific contributions to this phenotype have not been investigated systematically.

In the present study we use zebrafish, a biomedical and ecotoxicological research model organism [58,59], to further test the hypothesis that developmental FLX exposure results in effects on offspring endocrine stress axis. Zebrafish hold great promise not only to investigate long-term physiological effects of pharmaceuticals across developmental trajectories and the life-cycle, but also intergenerationally [60]. Zebrafish provide the additional advantage that molecular mechanisms of stress axis ontogeny [61,62] and developmental programming of the endocrine stress axis [63–66] are increasingly characterized. Finally, recent evidence shows that the endocrine stress axis in this model is responsive to FLX, as the endocrine stress axis activation following exposure to a mechanical stressor is dampened by acute and sub-chronic FLX exposure in adult zebrafish [41]. Within the framework of our hypothesis, we additionally sought to determine whether any intergenerational effects are related to a specific parental contribution, and whether life-stage (age and/or reproductive experience) may affect possible intergenerational effects of FLX on the endocrine stress axis. Finally, by probing known molecular transcripts related to endocrine stress axis linked to developmental programming of the stress axis in zebrafish [63–66], as well as epigenetic (*de novo* DNA methylation) and posttranscriptional (miRNA) regulation pathways linked to the intergenerational transmission of stress axis function in mammals [67,68], in gametes of unexposed control and developmentally FLX-exposed parents, we aimed to identify possible molecular mechanisms linked to the intergenerational inheritance of endocrine stress axis parameters in FLX-exposed zebrafish.

## 2. Materials and methods

### 2.1. Experimental design and animals

**Fig 1:**
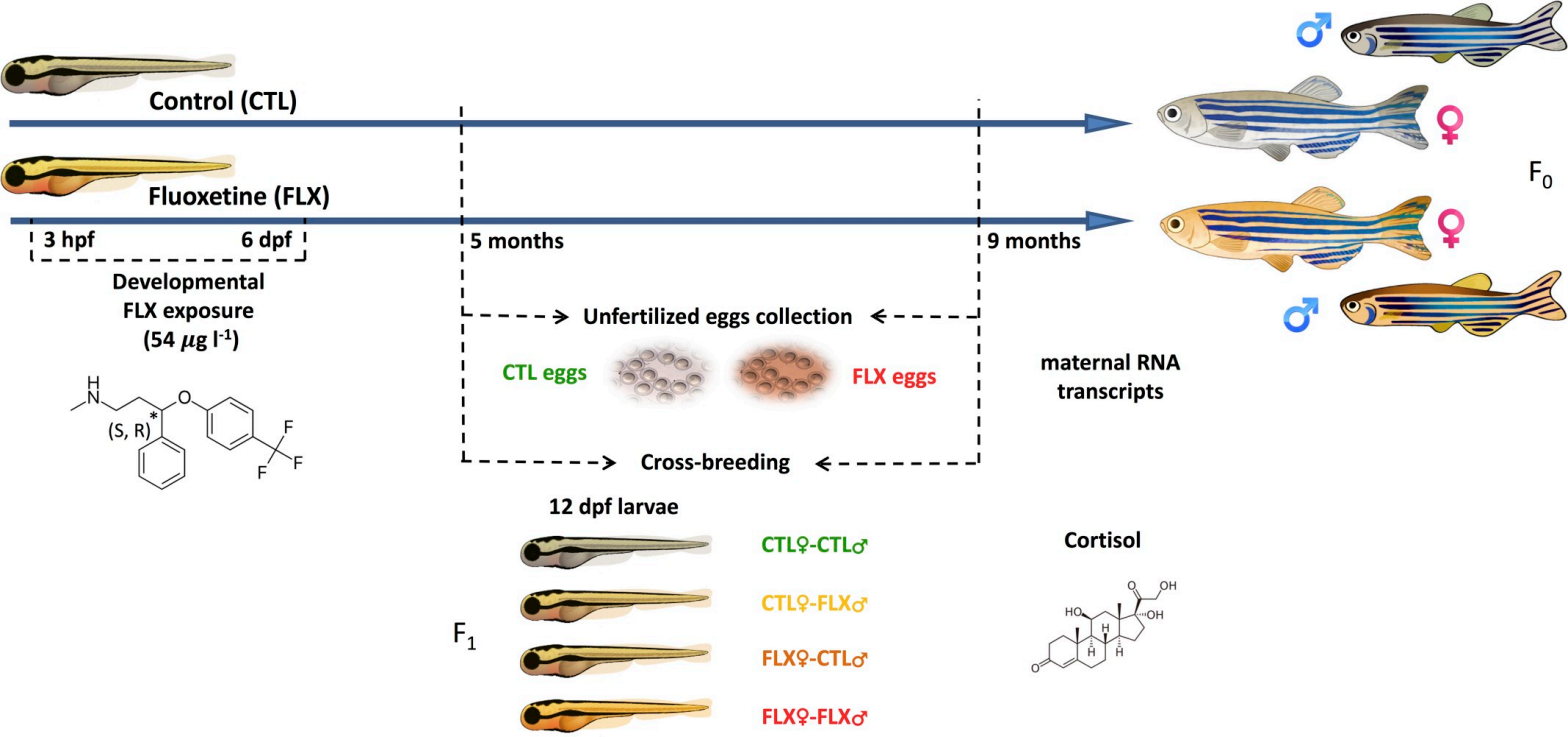
Schematic representation of the experimental design and measured endpoints.

Zebrafish embryos of the founder generation (F_0_) were obtained by mating 2 male and 4 female adult zebrafish (AB strain) in a 5L standalone plexiglass breeding tank (Aquatic Habitats, Apopka, FL, USA). Breeding groups were set up overnight in the tanks with an inner separator, dividing males from females overnight. This set-up allowed for olfactory and visual cues, but not physical interaction between the sexes. At 9:00 am the next morning, fish from all the groups were transferred to new tanks, halfway filled with fresh system water and the divider was subsequently removed to allow breeding for 1h 45min. Eggs were immediately collected and bleached (0.0075%) for 2 min, rinsed and divided into 2 groups: a control group (CTL), and a treatment group developmentally exposed to FLX (Sigma-Aldrich, Oakville, ON, Canada). Zebrafish embryos of both groups were reared in glass Petri dishes containing either embryo medium (E3) alone (CTL group) or E3 medium supplemented with a FLX stock solution for a final concentration of 54 μg/L of FLX solution (FLX group). The E3 medium was prepared diluting 20 ml of a 60x E3 medium stock in 980 ml of system water. The 60x E3 medium stock was composed of 34.8 g NaCl, 1.6 g KCl, 5.8 g CaCl_2_·2H_2_O and 9.78 g MgCl_2_·6H_2_O (all Sigma-Aldrich) dissolved in a total volume of 2 L of water, with a pH adjusted to 7.2. The exposure duration was between 3 hours post fertilization (hpf) and 6 days post fertilization (dpf) during which zebrafish embryos were maintained in an incubator (Thermo-Fisher Scientific, Ottawa, ON, Canada) at 28.5°C under constant darkness. Both CTL and FLX E3 media were changed daily to assure maintenance of the FLX concentration during the exposure period [13] and to remove debris and dead embryos. Following this developmental treatment period, all larvae were kept in E3 medium and fed with ZM Fry Food (Zebrafish Management Ltd., UK) of the appropriate size for their developmental stage. At 30 dpf, juvenile fish were transferred to tanks at a density of 5 fish/L in a flow-through system connected to aerated, dechlorinated city of Ottawa tap water maintained at 28.5°C. Thereafter, fish were fed three times daily with No. 1 crumble-Zeigler food (Aquatic Habitats). Following the developmental exposure period, larvae and adult zebrafish were housed under a 14 h light: 10 h dark photoperiod throughout the study. A summary of the developmental exposure groups is provided in **Fig 1**.

### 2.2. Breeding to obtain the F_1_ generation

At 5 months post fertilization (mpf), adult zebrafish (F_0_) were separated by sex following visual inspection of the pectoral fin breeding tubercles. Before breeding, fish were additionally fed with brine shrimp (*Artemia spp*.) for 10 min. Breeding took place under the same environmental conditions and procedures described above (section 2.1). Using adult CTL and FLX-exposed animals, four experimental groups were designed in a full factorial 2x2 cross-breeding design (summarized in **Fig 1**): 1) CTL females bred with CTL males (CTLF x CTLM), 2) CTL females bred with FLX-exposed males (CTLF x FLXM), 3) FLX-exposed females bred with CTL males (FLXF x CTLM), and 4), FLX females bred with FLX males (FLXF x FLXM). Seven replicate tanks were prepared for each treatment. Embryos (F_1_) were collected using a fine net, bleached for 2 min in 0.0075% bleach, and transferred to petri dishes filled with 50 ml of 1x embryo medium at a density of 35 embryos per dish. The embryo medium was changed every 2 days. Three cohorts of breeding trials were conducted. Larvae were kept at 28.5°C in an incubator, and fed ZM Fry food from 6 to 12 dpf, as previously described for F_0_. At 12 dpf, 8 replicates of 18–24 larvae pools per condition were snap-frozen and kept at -80°C for further basal cortisol analysis. Following the breeding at 5 mpf, fish were returned to the commonly housed pool of fish that were divided by sex and treatment history (developmental control or FLX exposure). At 9 mpf, a subset of fish was again randomly chosen from these pools for a second breeding trial following the same described protocol. All procedures conducted in this study were approved by the University of Ottawa Animal Care Protocol Review Committee and are in compliance with the guidelines of the Canadian Council on Animal Care for the use of animals in research.

### 2.3. Whole larvae lipid extraction and cortisol quantification

Total lipids (including cortisol) were extracted from 12 dpf larvae using a modified Folch protocol [69]: larvae were suspended in a total of 7.5 ml of Folch solution (CHCl_3_:MeOH, 2:1 v/v)) and sonicated. After a 15 min incubation period at room temperature, 2.5 ml of 2 M KCL with 5 mM EDTA were added to the homogenate, vortexed and incubated at room temperature for an additional 20 min. The organic layer at the bottom was then placed in a disposable culture tube and evaporated using nitrogen stream at 50°C. Lipids were subsequently resuspended in 0.1 ml of EGME (ethylene glycol monomethyl ether) and stored at -80°C for future whole larvae cortisol quantification. Cortisol concentrations were measured using a commercially available ELISA assay (Cayman chemicals, Ann Arbor, MI, USA), previously validated for zebrafish. Lipid samples suspended in EGME were diluted (1:4) in ELISA buffer from the same kit and the cortisol assay was performed following the manufacturer’s protocol. Measured cortisol levels were normalized to total protein concentrations from the same samples. Protein concentrations were obtained using a BCA (bicinchoninic acid) assay as per manufacturer’s protocol (Thermo-Fisher Scientific).

### 2.4. Adult tissue collection

After anaesthetizing 5 and 9 months old female fish of both CTL and FLX groups in a 1:50 diluted working solution of 3x Tricaine (1.4% tricaine solution, pH = 7), eggs were extracted from individual adult females by placing the fish in a Petri dish and gentle pushing on the abdomen. Eggs were then washed and suspended in 1x Hank’s solution, collected in 1.5 ml Eppendorf tube and immediately frozen at -80°C. 8 replicates per condition were collected (each of them consisting of eggs from an individual female). In a separate experiment using adult female CTL fish only (n = 15), ovaries, fins, and eggs were extracted in order to ascertain that egg extraction procedure does not result in transfer of RNA from ovary and/or anal fin to the egg. Following egg extraction as previously described, anal fins were cautiously clipped for collection, and ovaries dissected and stored. All tissues were rinsed in 1x Hank’s solution prior to storage at -80°C.

### 2.5. RNA extraction, reverse transcription and quantification of oocyte mRNAs

Total RNA was extracted using the Trizol method as described in the manufacturer’s protocol (Invitrogen, Oakville, ON, Canada). Extracted RNA was quantified and its purity assessed using a NanoDrop 2000c UV-Vis Spectrophotometer (Thermo-Fisher Scientific, Ottawa, ON, Canada). The cDNA was generated from 1 μg of extracted total RNA using a QuantiTech Reverse Transcription Kit (Qiagen, Toronto, ON, Canada) following the manufacturer’s protocol. A noRT negative control was also prepared from a sample pool by replacing the RT enzyme with water. Two step quantitative real-time polymerase chain reaction (qRT-PCR) was performed to assess relative fold changes in mRNA abundances of target genes between CTL and FLX group samples, using a CFX96 PCR machine (Bio-Rad, Mississauga, ON, Canada). Target genes included maternally deposited mRNAs coding for components of the endocrine stress axis (*pomca*, *pomcb*, *crhbp*, *gr*, *fkbp5*, *hsd11b2*), components of the miRNA biogenesis pathway involved in post-transcriptional control of gene expression (*dicer*, *drosha*, *dgcr8*, *xpo5*, *ago2*) and components of the epigenetic *de novo* DNA methylation pathway involved in transcriptional control of gene expression (*dnmt 3–8*). Specific primer sequences and annealing temperatures are listed in **Table 1**. All qPCR reactions were carried out in duplicate, using SsoAdvanced Universal Inhibitor-Tolerant SYBR Green Supermix (Bio-Rad, Mississauga, ON, Canada), following manufacturer’s protocol with reaction volume of 20 μl. Reaction parameters included an initial denaturation step at 95°C (20 s) and a combined annealing/extension step (30 s) over 40 cycles. The annealing temperatures for all primer pairs are listed in **Table 1**. Serial dilutions were used to generate standard curves for each specific primer pair, and acceptable parameters ranged between 100 ± 10% efficiency and an R^2^ value > 0.98. The negative control noRT was run with each assay to control for genomic DNA contamination. Following each assay, melting curves were systematically run and monitored for individual peaks. For the new unpublished primer set, resulting PCR products were purified using a Qiaquick PCR purification kit (Toronto, ON, Canada), and sequenced at the OHRI Stem Core laboratory (Ottawa, ON, Canada). Sequencing results were used in a BLAST search to confirm amplicon specificity. Gene expression data were normalized using the ΔΔCT method [70] using the geometric mean of *ef1a* and *β-actin* of each control group (5 or 9 months old), respectively.

**Table 1:** Primer sequences and reaction conditions of real-time RT-PCR to profile relative mRNAs abundance.

mRNA	Efficiency (%)	R^2^	Annealing T (°C)	size amplicon (nt)	NCBI ID	ENSEMBL ID	Primer FW	Primer RV
*Stress axis-related transcripts*								
*gr*	97.3	0.995	62	116	553740	ENSDARG00000025032	ACAGCTTCTTCCAGCCTCAG	CCGGTGTTCTCCTGTTTGAT
*fkbp5*	100.1	0.991	61	290	368924	ENSDARG00000028396	GCGAATCTCCCAGCTGTGTTTATC	GATCAAACGAACAAGCGGGTCTG
*pomca*	96.6	0.980	63	192	353221	ENSDARG00000043135	GCCCCTGAACAGATAGAGCC	CTCGTTATTTGCCAGCTCGC
*pomcb*	108.0	0.980	53	121	100034412	ENSDARG00000069307	TCCATCGAGCTCCAAAACCC	ACACTTTTACCGGTCTGCGT
*hsd11b2*	92.4	0.982	63	117	334098	ENSDARG00000001975	GGAGAGGGAGCCAAGCATTT	AAGTTTGGCCTTGGTGTCGA
*crhbp*	104.6	0.988	63	124	445065	ENSDARG00000024831	GGATAACGAGATCAGCCCGG	ACCCTCTACGGCCACCATAT
*Epigenetics / DNA methylation related transcripts*								
*dnmt3*	103.0	0.995	60	173	30659	ENSDARG00000057830	TAGAGTCATGTTGAACTGGGCC	TCAGGTCCAGAGATTCAGGGAT
*dnmt4*	92.6	0.990	60	141	317744	ENSDARG00000036791	AAGATTTACCCTGCAGTCCCAG	CTCGCATACTTCTGACGCAATG
*dnmt5*	98.4	0.996	60	143	323723	ENSDARG00000057863	TTATCCACCCACTGTTCGAAGG	ATGACCACACAGAATGACCTCC
*dnmt6*	94.6	0.987	60	200	553189	ENSDARG00000015566	GTGTGGGGAAAGTTACGAGGAT	TGCTTATTGTAGGTTGGCTGGT
*dnmt7*	90.9	0.991	60	174	321084	ENSDARG00000052402	AGGCAGCTTTTCGGGATTTAGA	CGATTTCTTGACCATCACGAGC
*dnmt8*	102.3	0.993	60	142	553187	ENSDARG00000005394	CTTTGCCTGTTAATGAAGCCCC	TGTGAAGTGTCCTGTGGTTGAA
*dicer1*	92.7	0.987	60	231	324724	ENSDARG00000001129	GCGACTCCTTCCTGAAACAC	TGTCTGTGCTGCTTTTGTCC
*drosha*	90.9	0.991	60	253	567505	ENSDARG00000055563	GGAGACCCGCAGTATCAAAA	TGTGATGGGTGAGAACAGGA
*Posttranscriptional regulation of gene expression / miRNA biogenesis- related transcripts*								
*xpo5*	101.5	0.982	60	293	558662	ENSDARG00000098868	TCACCATCGTCTCCACACTC	CTCCATGAGGGCACATTTCT
*dgcr8*	101.2	0.998	60	218	563963	ENSDARG00000035564	GTAGATGCCCTGTTGGAGGA	ACTGGAATGCCGGAGTTATG
*ago2*	109.1	0.992	60	114	570630	ENSDARG00000061268	TTACGTGCGTGAGTTTGGAG	GGGGTTGCTATTGCTTTGT
*ago2 (active)*	97.1	0.991	55	134	570630	ENSDARG00000061268	GGCAGTCACACATCAGGTCA	TTCAGGATTGTGGGGCTTGG
*actb1 (pair1)*	97.7	0.996	52	78	57934	ENSDARG00000037746	ACCATCGGCAATGAGCGTTT	GATACCGCAAGATTCCATACCCAG
*actb1 (pair2)*	93.0	0.995	53	98	57934	ENSDARG00000037746	CCCATCCATCGTTCACAGGA	CGAGAGTTTAGGTTGGTCGTTC
*ef1a*	94.3	0.998	57	169	30516	ENSDARG00000020850	AGATGCCGCCATTGTTGAGA	CTTTGTGACCTTGCCAGCAC

### 2.6. In silico prediction of zebrafish miRNAs-stress axis mRNA relationships

In order to identify miRNAs of interest to be quantified as potential molecular mediators of maternally deposited stress axis transcripts in transcriptionally silent oocytes, we first examined several transcriptomic datasets that have confirmed maternally deposited stress axis transcripts in zebrafish eggs [71–75]. Following confirmation of maternal presence, we examined recent transcriptomic datasets of zebrafish exposed to FLX to also identify whether transcripts are regulated by FLX exposure in zebrafish [51,53,55,76]. Finally, we used the available zebrafish TargetScan algorithm (*http*:*//www*.*targetscan*.*org/fish_62/*) to identify specific miRNAs predicted to bind the 3’UTR of the identified stress-related transcripts (**Table 2**). These miRNAs were then prioritized for quantification in eggs derived from CTL zebrafish, and eggs derived from zebrafish developmentally exposed to FLX.

**Table 2:** Target scan zebrafish derived identification of miRNAs that are expressed in unfertilized zebrafish egg and are predicted to target maternally deposited transcripts with function in the endocrine stress axis regulation. Several of these transcripts have been shown to respond to acute waterborne FLX exposure in zebrafish embryos.

miRNA	maternally deposited in unfertilized egg	3’UTR binding site number	8mer	7mer -m8	7mer -1A	Target Scan Score	miRNA conserved across vertebrates	targeted stress axis transcript	ENSEMBL ID	maternal deposition of stress transcript identified in unfertilized zebrafish egg		transcript regulated by acute FLX in zebrafish embryo
*dre-miR-740*	yes (current study)	1	0	0	1	-0.01	not conserved	*pomcb*	*ENSDARG00000069307*	yes (current study)	no	
*dre-miR-740*	yes (current study)	3	0	2	1	-0.05	not conserved	*pomca*	*ENSDARG00000043135*	yes (current study)	no	
*dre-miR-740*	yes (current study)	2	1	0	1	-0.11	not conserved	*hsd11b2*	*ENSDARG00000001975*	yes [74]	yes [58]	
*dre-miR-740*	yes (current study)	3	1	0	2	-0.05	not conserved	*gr*	*ENSDARG00000025032*	yes [74,2]	no	
*dre-miR-740*	yes (current study)	2	0	2	0	-0.04	not conserved	*fkbp5*	*ENSDARG00000028396*	yes [72, 74]	yes [56, 58,76]	
*dre-miR-740*	yes (current study)	3	2	0	1	-0.08	not conserved	*crhbp*	*ENSDARG00000024831*	yes [74,4]	no	
*dre-miR-30d*	yes [111]	1	1	0	0	-0.12	conserved	*hsd11b2*	*ENSDARG00000001975*	yes [74]	yes [58]	
*dre-miR-30d*	yes [111]	1	1	0	0	-0.22	conserved	*fkbp5*	*ENSDARG00000028396*	yes [72, 74]	yes [56, 58,76]	
*dre-miR-25/92a*	yes [111]	1	0	0	1	-0.09	conserved	*pomcb*	*ENSDARG00000069307*	yes (current study)	no	
*dre-miR-25/92a*	yes [111]	1	0	1	0	-0.06	conserved	*pomca*	*ENSDARG00000043135*	yes (current study)	no	
*dre-miR-25/92a*	yes [111]	1	0	1	0	-0.14	conserved	fkbp5	*ENSDARG00000028396*	yes [74]	yes [58]	
*dre-miR-26*	yes [111]	1	1	0	0	-0.03	conserved	*pomca*	*ENSDARG00000043135*	yes (current study)	no	
*dre-miR-26*	yes [111]	2	0	1	1	-0.23	conserved	*fkbp5*	*ENSDARG00000028396*	yes [74]	no	
*dre-miR-103*	yes [111]	2	0	1	1	-0.23	conserved	*fkbp5*	*ENSDARG00000028396*	yes [74]	no	

### 2.7. Relative quantification of miRNAs in adult tissues and oocytes

cDNAs for microRNAs was generated from the extracted total RNA using a miScript II reverse transcription kit (Qiagen), following the manufacturer’s protocol with 1 μg of total RNA as starting material for each reaction, and a noRT negative control. Specific miRNAs were then quantified using the miRScript SYBR Green PCR kit (Qiagen) with miRNA-specific forward primers and a universal reverse primer (**Table 3**). Reactions were run in duplicate on a CFX96 instrument (Bio-Rad, Mississauga, ON, Canada), with a total volume of 25 μl containing 2.5 μl of cDNA, 2.5 μl of 10 nM miRNA specific primer, 2.5 μl miScript Universal Primer, 12.5 μl of 2xQuantiTect SYBR Green PCR Master Mix (Qiagen), and 5 μl of H_2_O, according to the manufacturer’s instructions. For each assay, cycling parameters were an initial 15 min 95°C activation step, followed by 40 cycles of 15s incubation at 94°C, 30s at 60°C, and 30s at 70°C. After each run, melting curves were produced by a gradual increase in temperature from 65°C to 95°C in 0.5°C increments every 5s. The final melting curves were monitored for single peaks to confirm the specificity of the reaction and the absence of primer dimers. Standard curves and noRT controls were used to assess efficiency and specificity of amplifications as previously described. The ΔΔCT method for normalization [70] was adopted using *snoU23* as a reference gene, as previously described for rainbow trout [77]. The miRNA fold changes were then calculated relative to each CTL group (5 or 9 months old, in each case), as previously described. Additionally, to ensure the egg extraction procedure did not result in transfer of tissue-enriched miRNAs from ovary or fins to extracted eggs, we profiled *miRNA-181a* and *miRNA-143a* 5 month old control fish in egg, ovary and fin tissue.

**Table 3:** Primer sequences and reaction conditions of real-time RT PCR to profile microRNAs.

miRNA	Efficiency (%)	R^2^	Annealing T (°C)	size amplicon (nt)	miRBase ID	NCBI gene ID	Primer FW
*dre-miR-740*	101.5	0.987	51	22	MIMAT0003771	100033753	ATAAAAAGTGGTATGGTACAGT
*dre-mir-25-3p*	102.1	0.980	52	22	MIMAT0001793	100033594	CATTGCACTTGTCTCGGTCTGA
*dre-mir-26a-1-5p*	91.3	0.981	52	22	MIMAT0001794	100033595	TTCAAGTAATCCAGGATAGGCT
*dre-mir-30d-5p*	96.2	0.980	58	22	MIMAT0001806	100033612	TGTAAACATCCCCGACTGGAAG
*dre-mir-92a-1-3p*	97.8	0.983	59	22	MIMAT0001808	100033614	TATTGCACTTGTCCCGGCCTGT
*dre-mir-103-3p*	95.3	0.981	60	23	MIMAT0001816	100033625	AGCAGCATTGTACAGGGCTATGA
*dre-mir-181a-5p*	93.6	0.980	60	23	MIMAT0001623	100033461	AACATTCAACGCTGTCGGTGAGT
*dre-miR-143*	92.2	0.987	60	21	MIMAT0001840	100033666	TGAGATGAAGCACTGTAGCTC
*dre-snoU23*	91.0	0.988	53	23	AJ009730	-	GCCCATGTCTGCTGTGAAACAAT

### 2.8. Data analyses

In all cases, data were analyzed for normality and homoscedasticity using Shapiro-Wilk test and Levene’s test, respectively, to ascertain that ANOVA and/or t-test criteria were met. In cases were data did not meet these criteria even after transformation, equivalent non-parametric tests (Kruskal-Wallis, Mann-Whitney U test) were used. In cases data was normally distributed, Grubb’s test was performed to identify outliers. All statistical analysis and graphing procedures were performed using SPSS Version 24.0 (Armonk, NY: IBM Corp., 2016) and Prism Software, Version 7 (Graphpad, Irvine, CA, USA). Whole larvae body cortisol data were analyzed by individual 2-way ANOVAs for each breeding timepoint, using maternal and paternal exposure as main factors, and a subsequent Tukey’s post-hoc analysis was conducted with cut of p < 0.05.

All gene expression data were analyzed with unpaired t-test or Mann-Whitney U tests, with a significance level of p<0.05. Principal component analysis (PCA) and hierarchical clustering/heatmaps were performed across all samples using as variables the relative abundance of all measured transcripts (and miRNAs) and graphed in R (www.R-project.org) with the packages *stats*, *factoextra*, *gplots* and *pheatmap*.

## 3.Results

### 3.1. Basal body cortisol levels in F_1_ larvae

**Fig 2:**
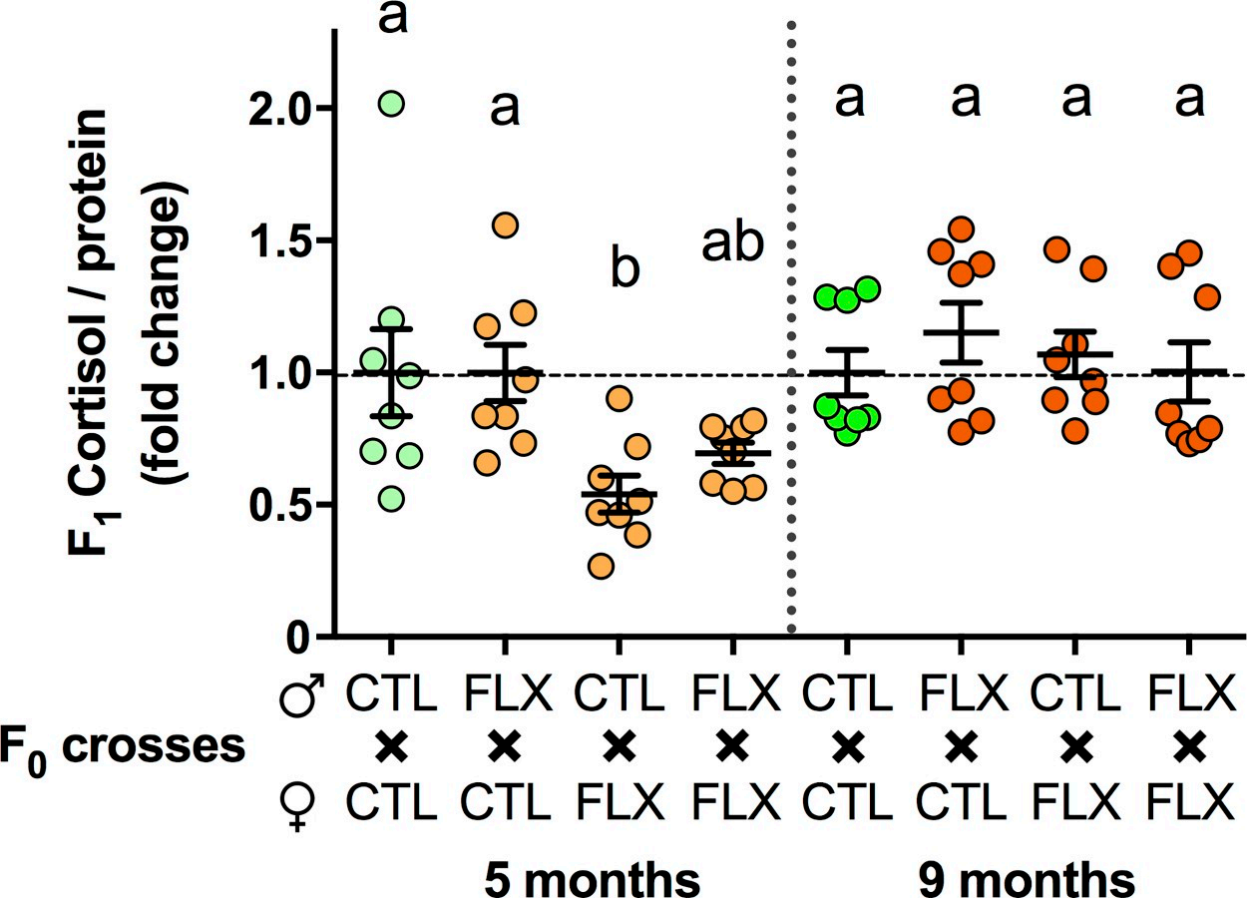
Basal body cortisol levels in whole F_1_ zebrafish larvae at 12 dpf. Larvae were generated by cross-breeding male and female fish that were either unexposed or exposed to 54 μg/L FLX between 0–6 dpf, generating four possible crosses. Larvae were generated using naive breeding pairs aged 5 months and the experiment repeated at 9 months. Cortisol assays were performed on Folch-extracted lipids of n = 8 pools of 18–24 zebrafish, and normalized to total protein levels determined by BSA assay. Values were normalized to respective unexposed breeding pairs, and individual data points, mean values and S.E.M. are presented. Data was not normally distributed and analyzed using non-parametric Kruskal-Wallis tests to identify maternal and paternal contribution, as well as differences between four crosses at a given breeding time. A p<0.05 was used as cut-off and letters indicate significant differences between groups.

A significant maternal (df = 1, H = 11.251, p<0.001), but not paternal (df = 1, H = 1.455, p = 0.228) effect on whole body basal cortisol levels were evident in 12 dpf old F_1_ larvae obtained from mating all four different combinations of 5 mpf F_0_ females and males with CTL or developmental FLX exposure history. Significant decreases in cortisol concentrations were found in larvae derived from F_0_ females with developmental FLX exposure history and unexposed F_0_ males compared to controls (df = 3, H = 13.026, p<0.005, **Fig 2**). Conversely, 12 dpf old F_1_ offspring derived from mating 9 month old F_0_ females and males with unexposed control or developmental FLX exposure histories revealed neither maternal (df = 1, H = 1.396, p = 0.696) nor paternal (df = 1, H = 0.172, p = 0.970) contributions to basal cortisol concentrations, and no difference between the four treatment groups could be detected (df = 3, H = 3.179, p = 0.365; **Fig 2**).

### 3.2. Abundance of stress axis transcripts in zebrafish egg

**Fig 3:**
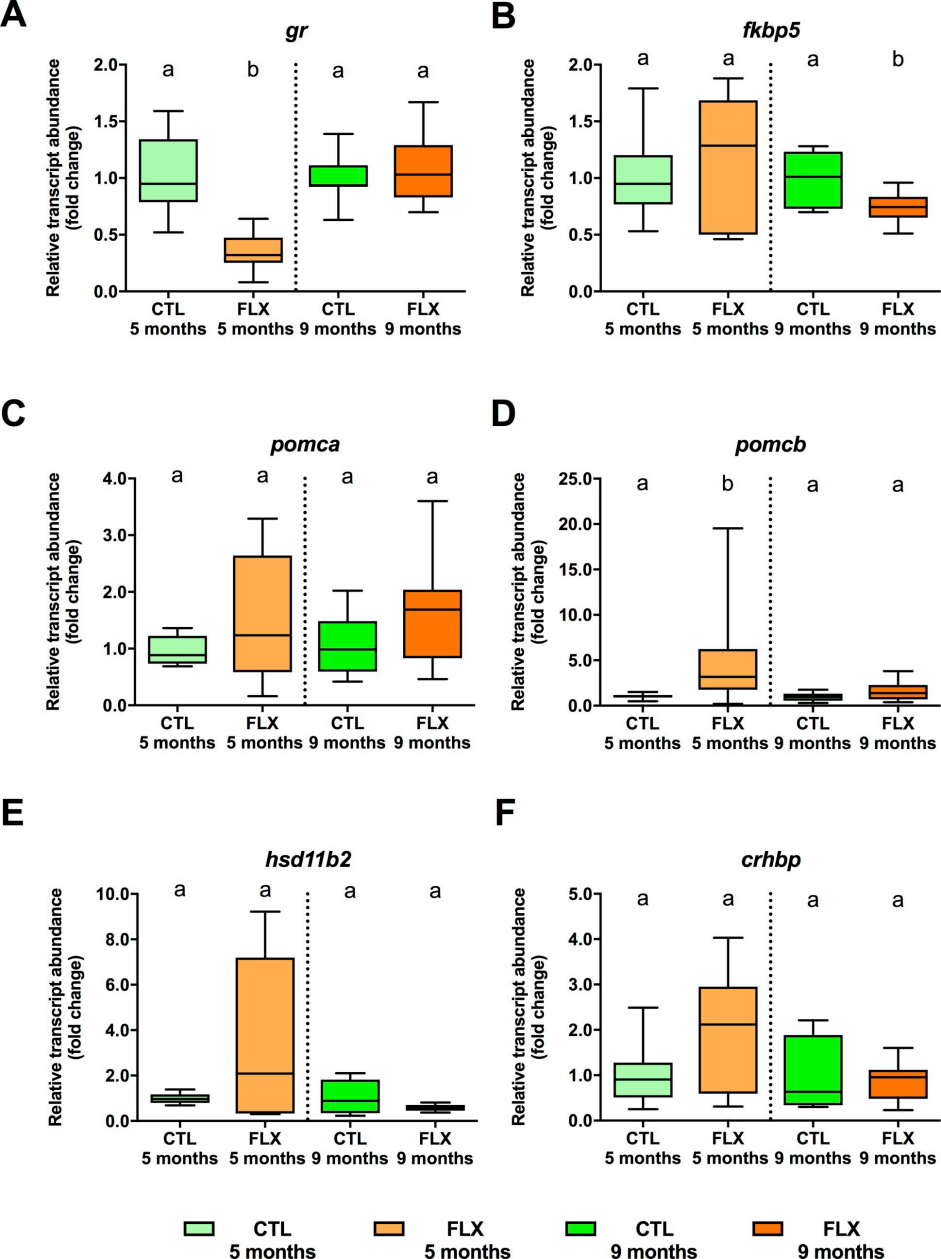
Steady-state abundance of maternal transcripts related to endocrine stress axis in eggs derived from 5 and 9 months old zebrafish. An n = 8 samples of pooled eggs extracted from a single female fish (CTL and FLX groups) were used to quantify the glucocorticoid receptor *gr* (**A**), the glucocorticoid modulator *fkbp5* (**B**) pro-opiomelanocortin a *pomca* (**C**), pro-opiomelanocortin b *pomcb* (**D**), 11*β*-hydroxysteroid dehydrogenase 2 *11bhsd2* (**E**) and the corticotropin releasing hormone binding protein *crhbp* (**F**). Data are normalized to respective control groups at 5 and months, and expressed as fold-change. Data were analyzed using t-test or Mann-Whitney U test, and significant differences (p<0.05) are indicated by different letters within each sampling timepoint.

Abundance of several stress axis-related transcripts in unfertilized eggs (the maternally-derived germline) changed significantly between treatment groups (**Fig 3**). With regard to the glucocorticoid receptor *gr* (**Fig 3A**), we found a reduction in transcript abundance in eggs collected from 5 months old females which were developmentally exposed to FLX (F = 20.102, p = 0.001), but not in eggs collected from FLX females at 9 months (df = 1, F = 0.259, p = 0.619). No changes in the glucocorticoid receptor modulator *fkbp5* transcript abundance (**Fig 3B**) were detected between CTL and FLX group eggs at 5 months (df = 1, F = 0.056, p = 0.817), but its abundance was increased in the FLX group eggs at 9 months compared to the CTL group (df = 1, F = 5.523, p = 0.034). Inversely, pro-opiomelanocortin b, *pomcb* transcript abundance (**Fig 3D**), was increased in eggs of the FLX group compared to CTL eggs collected at 5 months (df = 1, F = 4.848, p = 0.045), but not 9 months (df = 1, F = 1.234, p = 0.285). No significant changes were observed between eggs derived from CTL or FLX groups in the oocyte transcript abundance of the adrenocorticotropic hormone precursor pro-opiomelanocortin a (*pomca*), 11*β*-hydroxysteroid dehydrogenase 2 (*11bhsd2*) and corticotropin releasing hormone binding protein (*crhbp*) at 5 months (**Fig 3C,** df = 1, F = 0.186, p = 0.673; **Fig 3E,** df = 1, F = 0.766, p = 0.396; **Fig 3F,** df = 1, F = 1.942, p = 0.185) or 9 months (**Fig 3C,** df = 1, F = 1.894, p = 0.190; **Fig 3E,** df = 1, F = 0.976, p = 0.340; **Fig 3F,** df = 1, F = 0.04, p = 0.953).

### 3.3. Abundance of transcripts involved in DNA methylation

**Fig 4:**
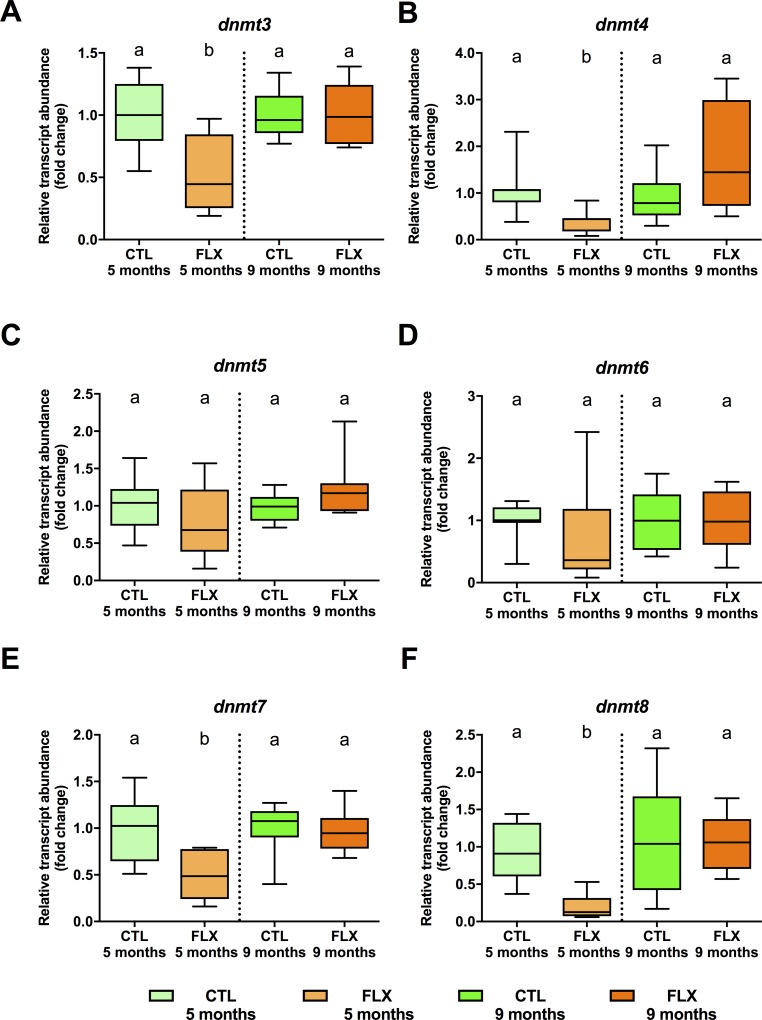
Steady-state abundance of maternal transcripts related to de novo DNA methylation in eggs derived from 5 and 9 months old zebrafish. An n = 8 samples of pooled eggs extracted from a single female fish (CTL and FLX groups) were used to quantify DNA methyltransferases *dnmt3* (**A**), *dnmt4* (**B**), *dnmt5* (**C**), *dnmt6* (**D**), *dnmt7* (**E**), and *dnmt8* (**F**). Data are normalized to respective control groups at 5 and months, and expressed as fold-change. Data were analyzed using t-test or Mann-Whitney U test, and significant differences are indicated by different letters within each sampling timepoint.

Abundance of some of the *de novo* DNA methyl-transferase (*dnmt*) paralogues transcripts (**Fig 4A–4F**) were significantly affected by developmental FLX treatment in oocytes collected at 5 months, with reductions of *dnmt3* (df = 1, F = 9.759, p = 0.007; **Fig 4A**), *dnmt4* (df = 1, F = 12.776, p = 0.003; **Fig 4B**), *dnmt7* (df = 1, F = 9.346 p = 0.009; **Fig 4E**), and *dnmt8* (df = 1, F = 30.744, p = 0.001; **Fig 4F**). Conversely, *dnmt5* (df = 1, F = 1.836, p = 0.197; **Fig 4C**) and *dnmt6* (df = 1, F = 3.301, p = 0.0091; **Fig 4D)** transcript abundance did not exhibit any statistically significant differences. No significant differences in the transcript abundance of any of the *dnmt* paralogues were observed between groups collected at 9 months (**Fig 4A–4F**).

### 3.4. Abundance of transcripts involved in miRNA biogenesis

**Fig 5:**
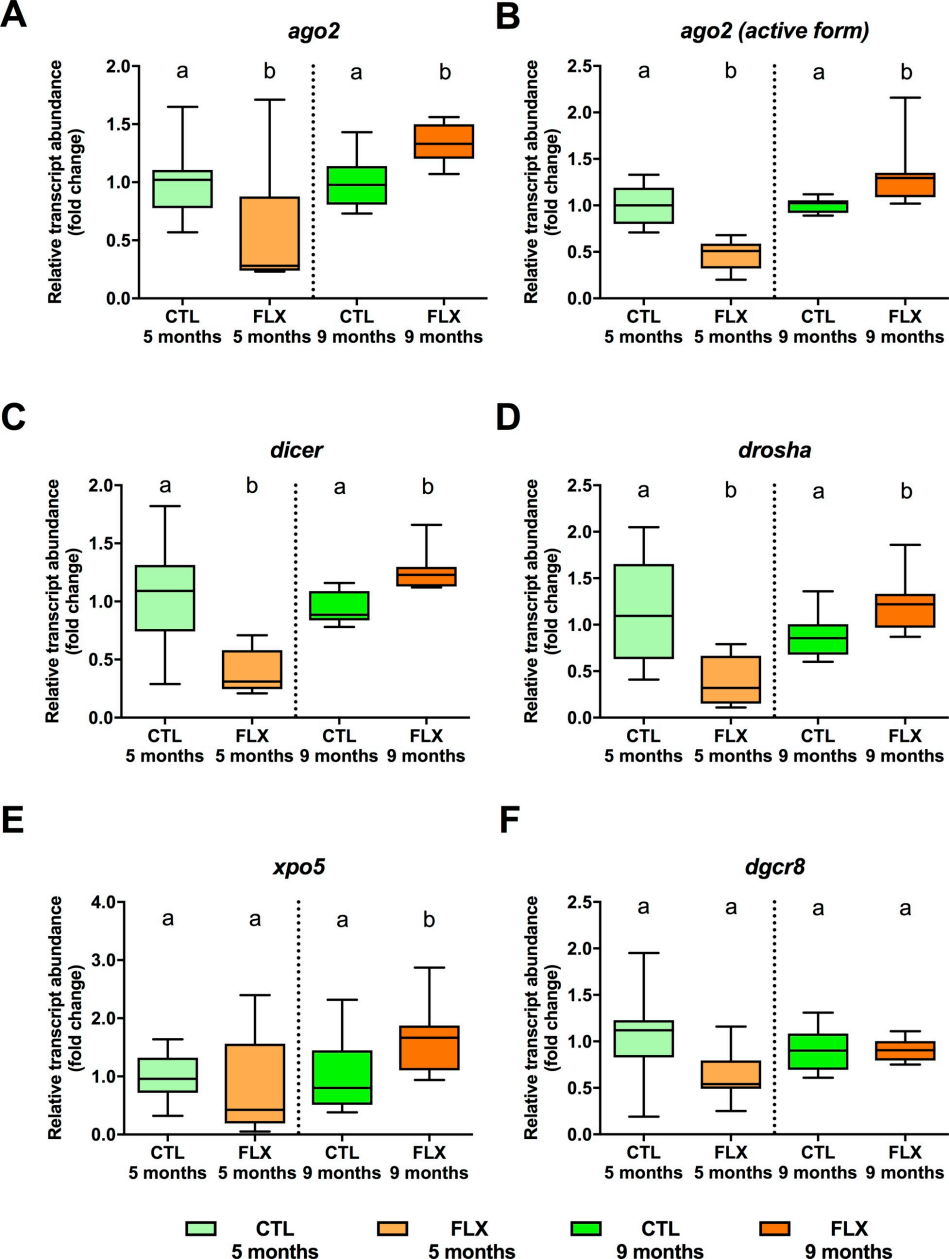
Steady-state abundance of maternal transcripts related to miRNA biogenesis and function in eggs derived from 5 and 9 months old zebrafish. An n = 8 samples of pooled eggs extracted from a single female fish (CTL and FLX groups) were used to quantify argonaute 2 protein *ago2* (**A**), the active *ago2* form containing the catalytic site (**B**), *dicer* (**C**), *drosha* (**D**), exportin 5 *xpo5* (**E**), and pasha *dgcr8* (**F**). Data are normalized to respective control groups at 5 and 9 months, and expressed as fold-change. Data were analyzed using t-test or Mann-Whitney U test, and significant differences are indicated by different letters within each sampling timepoint.

Oocyte transcripts of components of the miRNA biogenesis pathway (**Fig 5A–5F**), specifically the argonaute RISC catalytic component *ago2* (df = 1, F = 8.873, p = 0.010, **Fig 5A**) and its active form (df = 1, F = 23.248, p = 0.001; **Fig 5B**) and the type III ribonucleases *dicer* (df = 1, H = 16.016, p = 0.001 **Fig 5C**) *and drosha* (df = 1, F = 12.205, p = 0.004; **Fig 5D**) were decreased in eggs collected from FLX exposed females at 5 months. Conversely, at 9 months, *ago2* (df = 1, F = 12.038, p = 0.004; **Fig 5A**) and its active form (df = 1, F = 9.429, p = 0.008, **Fig 5B**), *dicer* (df = 1, H = 18.513, p = 0.001; **Fig 5C**), *drosha* (df = 1, F = 7.448, p = 0.16, **Fig 5D)**, and the exportin *xpo5* (df = 1, F = 5.247, p = 0.035; **Fig 5E**) transcript abundance were increased in eggs collected from FLX-exposed females. No significant changes were observed in eggs collected from FLX exposed females on transcript abundance of the microprocessor complex subunit *dgcr8* at 5 months (df = 1, F = 2.909, p = 0.110; **Fig 5F**) or 9 months (df = 1, F = 0.008, p = 0.931; **Fig 5F**).

### 3.5. Expression of miRNA in egg tissue

**Fig 6:**
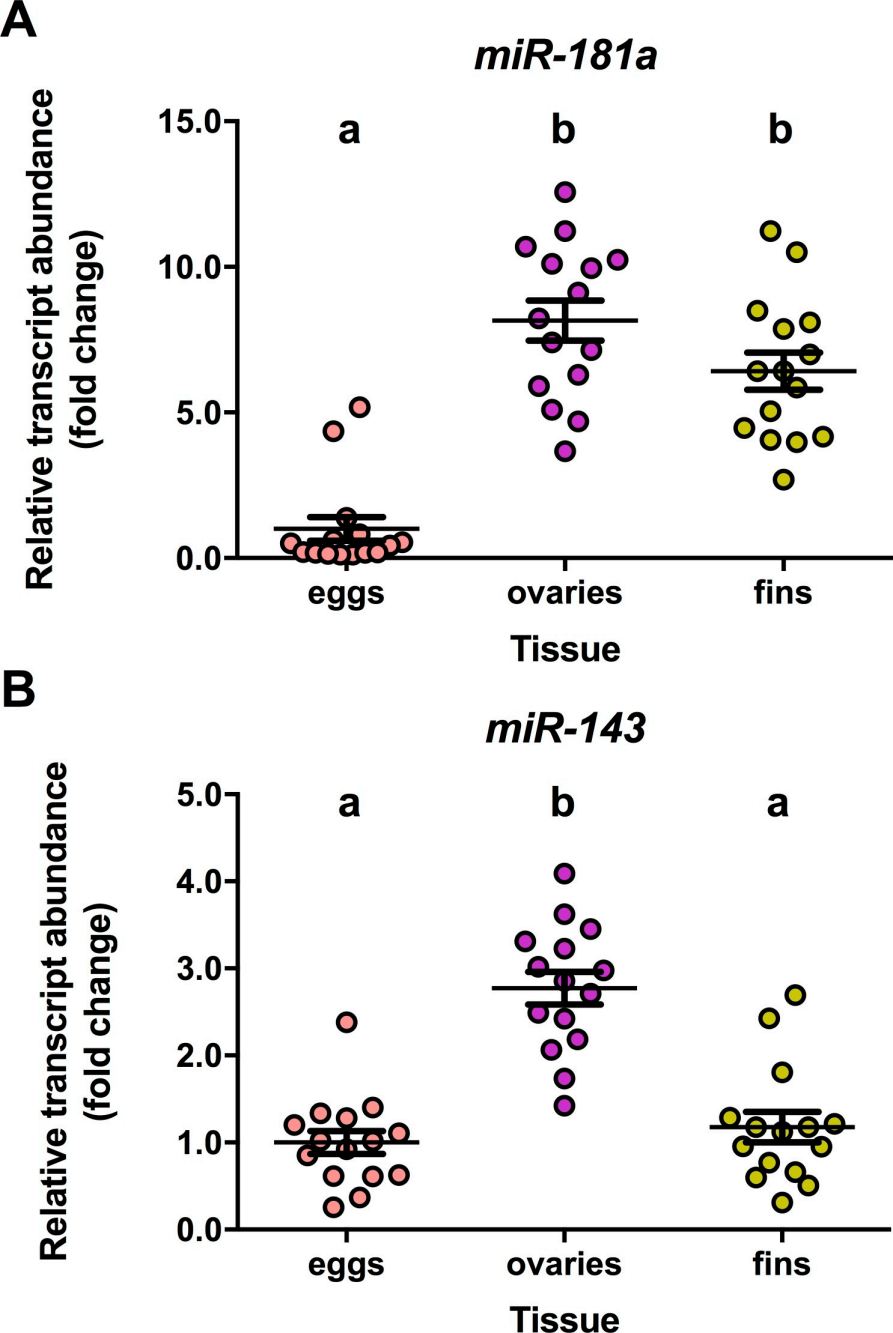
Steady-state abundance of tissue-enriched miRNAs [111] to ascertain that oocyte extraction procedure does not cause contamination by ovary or fin tissues (see text for explanations). An n = 13–15 samples of pooled eggs, ovaries tissue not containing extractable eggs, anal fin were collected from a single female fish (CTL and FLX groups), and were used to quantify *miRNA-181a* (**A**) and *miRNA-143* (**B**). Data are normalized to *sno-U23* RNA abundance and subsequently expressed as fold changed compared to egg miRNA abundance. Data were analyzed using one- way ANOVA, and significant differences resolved by Tukey’s post-hoc test are indicated by different letters.

In our preliminary miRNA profiling experiments to address possible transfer of tissue-enriched miRNAs from ovary and fin tissue to eggs during the extraction process, the abundance of *miR-181a* (df = 2, F = 37.71, p = 0.001) was significantly higher (p<0.01) in ovary and fin tissue compared to egg (**Fig 6A)** while abundance of *miRNA-143* was significantly higher (p<0.01) in ovaries compared to eggs and fins (df = 2, F = 32.04, p = 0.001; **Fig 6B)**. These expression results reveal that the extraction procedure itself did not result in transfer of miRNA between tissues and eggs.

**Fig 7:**
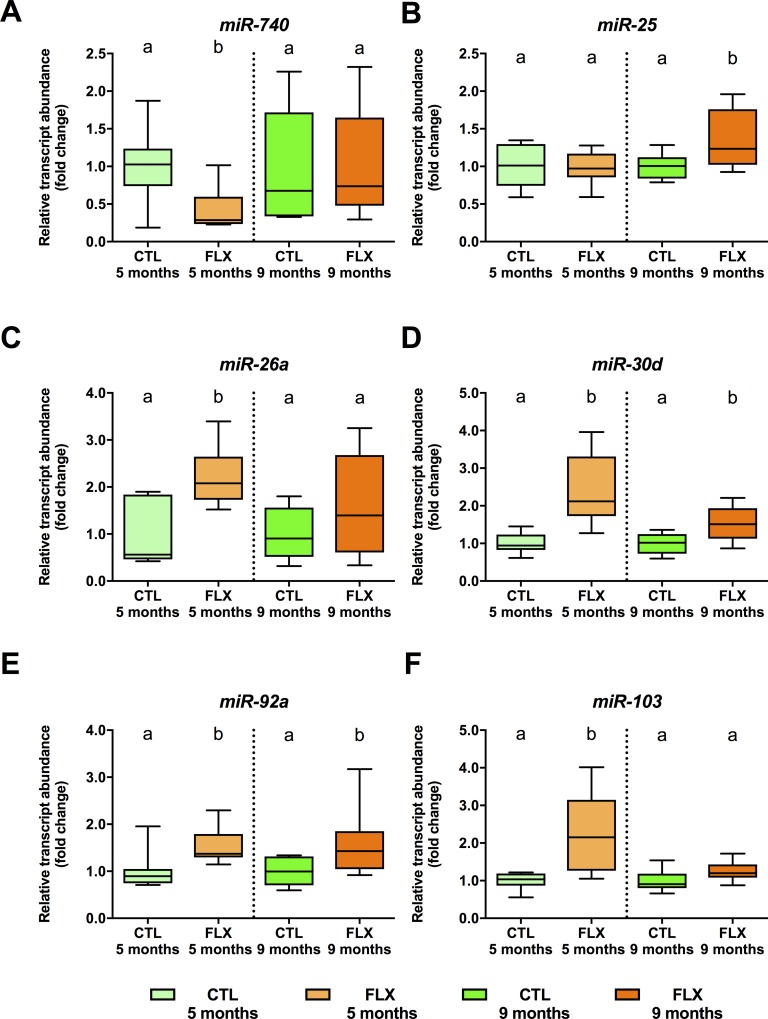
Steady-state abundance of maternal miRNAs predicted to target specific stress transcripts in eggs derived from 5 and 9 months old zebrafish. An n = 8 samples of pooled eggs extracted from a single female fish (CTL and FLX groups) were used to quantify *miRNA-740* (**A**), *miR-25* (**B**), *miR-26a* (**C**), *miR-30d* (**D**), *miRNA-92a* (**E**) and *miRNA-103* (**F**). Data are normalized to respective control groups at 5 and 9 months, and expressed as fold-change. Data were analyzed using t-test or Mann-Whitney U test, and significant differences are indicated by different letters within each sampling timepoint.

The expression of several miRNAs predicted to target stress axis transcripts (**Table 3**) was assessed in eggs of both CTL and FLX groups. Abundance of *miRNA-740* (**Fig 7A**) was significantly decreased by FLX in eggs collected at 5 months (df = 1, F = 7.134, p = 0.018), but not 9 months (df = 1, F = 0.042, p = 0.841). Abundance of *miRNA-25* (**Fig 7B**), was unchanged in eggs collected at 5 months (df = 1, F = 0.001, p = 0.973), but significantly increased in eggs collected from developmentally FLX-exposed females compared to CTL group eggs (df = 1, F = 5.913, p = 0.029). Maternal developmental FLX exposure resulted in increased oocyte abundance of *miRNA-26a* (df = 1, F = 13.843, p = 0.002; **Fig 7C**), *miRNA-30d* (F = 24.752, p = 0.001; **Fig 7D**), *miRNA-92a* (df = 1, F = 10.404, p = 0.006; **Fig 7E**), *miRNA-103* (df = 1, F = 14.829, p = 0.002) at 5 months (**Fig 7F**). In the case of *miRNA-30d* (df = 1, F = 7.524, p = 0.016; **Fig 7D**), and *miRNA-92* (df = 1, F = 5.336, p = 0.037; **Fig 7E**), increased abundance in FLX-female derived eggs was also observed in eggs collected at 9 months.

## 4. Discussion

### 4.1. Developmental exposure to FLX results in maternal transmission of reduced basal cortisol that is dependent on breeding experience and/or age

It has recently been shown in adult zebrafish that whole body cortisol levels in response to a stressor are suppressed in fish exposed to FLX [41,78], reflecting previously described phenotypes in rodents [79]. Indeed, a reduction of cortisol in response to FLX has been implicated as potential mechanism in the efficacy of FLX as antidepressant, owing to the fact that a dysregulation of the endocrine stress axis may underlie the etiology of depression [79]. It is generally believed that FLX alters the stress axis via its principal target, the serotonin transporter, through which serotonin concentration in the synaptic cleft is enhanced, altering serotonin signaling via postsynaptic receptor in a time-dependent manner [40,80]. FLX, at the concentration of 54 μg/L used in the present study is in the range human therapeutic dose range in fish [81], and similar concentrations of FLX have been shown to elicit serotonin turnover changes in fish brains [22,46]. Serotonin regulates the stress axis in fish [82–84], as in mammals [23]. Given that FLX interacts with specific CYP enzymes in fish [85], and in mammals [1], it is possible that altered hepatic cortisol clearance may also contribute to the stress axis regulation in response to FLX, although this has not been formally tested. Irrespective of the mechanism, it is clear that in both zebrafish and other fish species, as well as rodent models, FLX similarly affects the central serotonin and endocrine stress axis with evidence for time- dependent effects, likely reflecting desensitization events at the receptor level [39,40,42,46].

Our study extends the effect of developmental FLX exposure on the endocrine stress axis to offspring. In our current experiment, zebrafish were developmentally exposed to FLX between 0 to 6 dpf, a period which comprises germ-cell migration [86], organogenesis [87] and functional endocrine stress axis development [61,62]. Serotonin, in this period, is considered an important regulator of motor neuron differentiation and locomotion in developing zebrafish [88,89], but whether developmental serotonin acts as a programmer of the endocrine stress axis in zebrafish has not been directly investigated. Irrespective of an involvement of developmental serotonin, a recent study [57] was first to show that developmental exposure to FLX (both exposed F_0_ males and females) reduces F_0_ baseline and mechanical stressor-induced whole-body cortisol levels, and that this phenotype can be transmitted in subsequent generations, especially in males. We here additionally determine that this developmental exposure to FLX in the F_0_ generation results in maternal, but not paternal transmission of reduced basal cortisol to F_1_ offspring generated from crosses of 5 months old, but not 9 months old F_0_ zebrafish. Together, these results indicate a specific maternal contribution and life stage-dependency of the intergenerational suppression of an index of the endocrine stress axis in response to ancestral FLX exposure.

Our data agree with several studies describing effects of maternal FLX exposure on the endocrine stress axis in offspring described in mammals [90–92], although these studies investigated acute offspring exposure to FLX in the perinatal period which includes windows for placental transfer and/or lactation in mammals, and therefore may be indicative of more direct FLX effects on F_1_. Indeed, because the half-life of FLX in fish is in the range of days [13], our data show that F_1_ offspring is not directly exposed to FLX other than via primordial migrating germ cells [86] in gonadally undifferentiated [93] F_0_ zebrafish exposed to FLX at 0–6 dpf. This suggests that the maternal transmission is dependent on indirect mechanisms of FLX in eggs, which can be mediated, for example, by altered F_0_ stress axis function and cortisol deposition in eggs linked to endocrine stress axis programming in zebrafish [65,66] or (epigenetically regulated) transcript alterations in female germ cells. Zebrafish, contrary to rodent models, do not display maternal care behaviour, which is well-described to affect the mammalian stress axis [94], and has recently been shown to be affected by direct FLX administration in dams [95]. Therefore, the observed maternal transmission of FLX effects on the endocrine stress axis in zebrafish eliminates maternal behavior as confounding factor [60], and also allows us to investigate potential molecular mechanisms in eggs independently of maternal behavior effects. In contrast to the described maternal effects, we did not identify paternal contributions on offspring endocrine stress axis. While SSRIs, including FLX, are known to affect sperm quality and DNA integrity in humans [96], paternal effects of FLX exposure on the stress axis have, to our knowledge, never been investigated, in spite of the observation that sperm is an important mediator of inheritance in response to parental stress experience at least in mammals [67], an effect that has been causally linked to molecular epigenetics, especially miRNAs [68]. Our study shows that, at least in zebrafish, paternal developmental exposure to FLX does not play a role in altering the basal cortisol level index of the offspring endocrine stress axis, suggesting that paternal developmental SSRI exposure has limited effects on offspring stress axis functioning in this model. However, future studies should also investigate the possibility that acute parental FLX exposures may differentially affect offspring stress axis compared to our developmental parental exposure protocol employed here.

A second aspect of our experimental design tested the hypothesis that specific parental inheritance of basal cortisol is dependent on life-stage, which can be related to reproductive experience and/or age. Since most of the studies that investigate the inter and/or trans-generational effects of exposure to environmental contaminants in zebrafish and rodents are conducted in sexually naïve males or females, it is uncertain if breeding experiences and/or age may be a potential factor of this transgenerational transmission [60,97]. However, while largely unstudied [60], the question of whether intergenerational transmission of is dependent on age and/or breeding experience is an important one, as fitness in a majority of species, including asynchronously breeding zebrafish, is not mediated by a single reproductive event. Here we show that crossing developmentally exposed zebrafish at 9 months does not result in transmission of altered basal cortisol levels observed at 5 months, suggesting that maternal inheritance of this larval F_1_ phenotype is dependent on F_0_ breeding experience/and or age. To our knowledge, the only study investigating the effects of FLX on stress endpoints in relation to breeding experience has exclusively focused on Sprague-Dawley rat dams, but not offspring [95]. Interestingly, FLX blunted stress-induced corticosterone concentrations in nulliparae only, but not post-partum rats, suggesting differential action of FLX based on reproductive experience in this rat model. Age-dependent effects of FLX have been reported on stress axis related behaviors [98], and the role of FLX on several endpoints including the endocrine stress axis may be linked to sensitizing the organism to the living environment which differs between timepoints [99]. However, there are currently no studies investigating whether parental breeding history and/or age modulate effects of FLX on the offspring endocrine stress axis. Because our study randomly sampled F_0_ males and females for breeding at 5 and 9 mpf from maintained pools of CTL and FLX fish, we are unable to trace individual mating history and can consequently not distinguish between breeding experience and age-dependent effects. Future studies should therefore further dissociate these factors by separate breeding designs accounting for each factor individually.

Interestingly, in several fish species [100], and zebrafish in particular [65,101,102], maternal cortisol deposition in embryos has been postulated to be a nongenomic mechanism to program the endocrine stress axis. Our study cannot dissociate whether the maternal and life-stage specific inheritance of decreased baseline cortisol is dependent on indirect programming of the stress axis via differential F_0_ cortisol deposition in the eggs and/or via epigenetic programming of (primordial) germ cells, and future studies should investigate egg cortisol deposition.

### 4.2. Developmental FLX exposure alters the molecular signature in oocytes extracted from 5, but not 9 months old females

In order to investigate possible (epigenetic) molecular components involved in the maternally transmitted, life-stage dependent reduction of basal cortisol concentrations in the F_1_ larvae, we probed abundance of maternally deposited transcripts in unfertilized eggs collected from CTL and FLX F_0_ females at 5 and 9 mpf. We specifically measured the abundance of transcripts involved in the stress axis, in epigenetic regulation of gene expression (*de novo* methyltransferases and miRNA biogenesis components), as well as specific miRNA transcripts predicted to target stress axis transcripts. With regard to stress axis transcripts, we identified a significant reduction of *gr* and an induction of *pomcb* in FLX-group eggs collected at 5 mpf compared to controls, and a reduction of *fkpb5* in eggs collected in the FLX group compared to the control group at 9 mpf. Interestingly, Gr signaling has been shown to be important in zebrafish stress axis programming: Embryonic modulation of Gr function via *gr* morpholino from 2–8 cell stage to 120 hpf stage, which spans both maternal transcripts and *gr* transcripts synthesized following zygotic genome activation (ZGA) at mid-blastula stage (512–1024 cell stage at ~2–3 hpf [71], resulted in reduced whole larvae basal cortisol concentrations at 120 hpf, an effect also observed in response to the Gr agonist dexamethasone [63]. Modulation of maternal Gr signaling by cortisol injection or injection of a cortisol sequestering antibody in single cell embryos resulted in contrasting effects in baseline cortisol in 72 hpf larvae, with increases in response to cortisol treatment and decreases in response to cortisol AB treatment [65]. Conversely, following exposure to a mechanical stressor, cortisol increase is blunted in larvae developed from cortisol injected eggs and heightened in larvae developed from eggs injected with a cortisol sequestering antibody [65]. Together, these studies show a role for maternal Gr signaling in zebrafish offspring stress axis development. Therefore, the decrease observed in *gr* transcripts in FLX eggs collected at 5 months in the present study may suggest a causal role for the observed reduction in basal cortisol levels in offspring reared from developmentally exposed F_0_ females. Future rescue experiments using *gr* mRNA injection in single cell embryos from developmentally FLX-exposed F_0_ females are warranted to probe causality.

With regard to other maternally-deposited stress axis transcripts, we conversely identified significant increases in *pomcb* and directional increases in other stress axis transcripts in FLX eggs compared to CTL eggs collected from F_0_ at 5 mpf. Because of comparatively large variability, these directional increases in FLX eggs do to not reach statistical significance compared to CTL eggs. Interestingly, decreased *pomca* and *pomcb* transcript abundances are observed in 5 dpf *gr* ^-/-^ zebrafish larvae, suggesting that these transcripts are repressed via Gr [103]. In eggs collected from F_0_ at 9 mpf, a significant reduction in *fkpb5*, a chaperone of Gr that is cortisol responsive and diminishes cortisol binding to Gr in an ultrashort feedback loop [104], is reduced in FLX group eggs, suggesting possibly increased Gr signaling. Interestingly, *fkbp5* has consistently emerged as being regulated in transcriptomic screens of zebrafish embryos acutely exposed to FLX (**Table 2**). Together, our findings reveal for the first time, that developmental exposure of FLX results in differential maternal deposition of transcripts with described roles in programming the offspring’s endocrine stress axis. While both nongenomic factors, such as cortisol deposition, or epigenetic changes in germ cells may be responsible for this regulation, these data point to a possible mechanism in the maternal inheritance of reduced basal cortisol in response to parental developmental FLX exposure.

**Fig 8:**
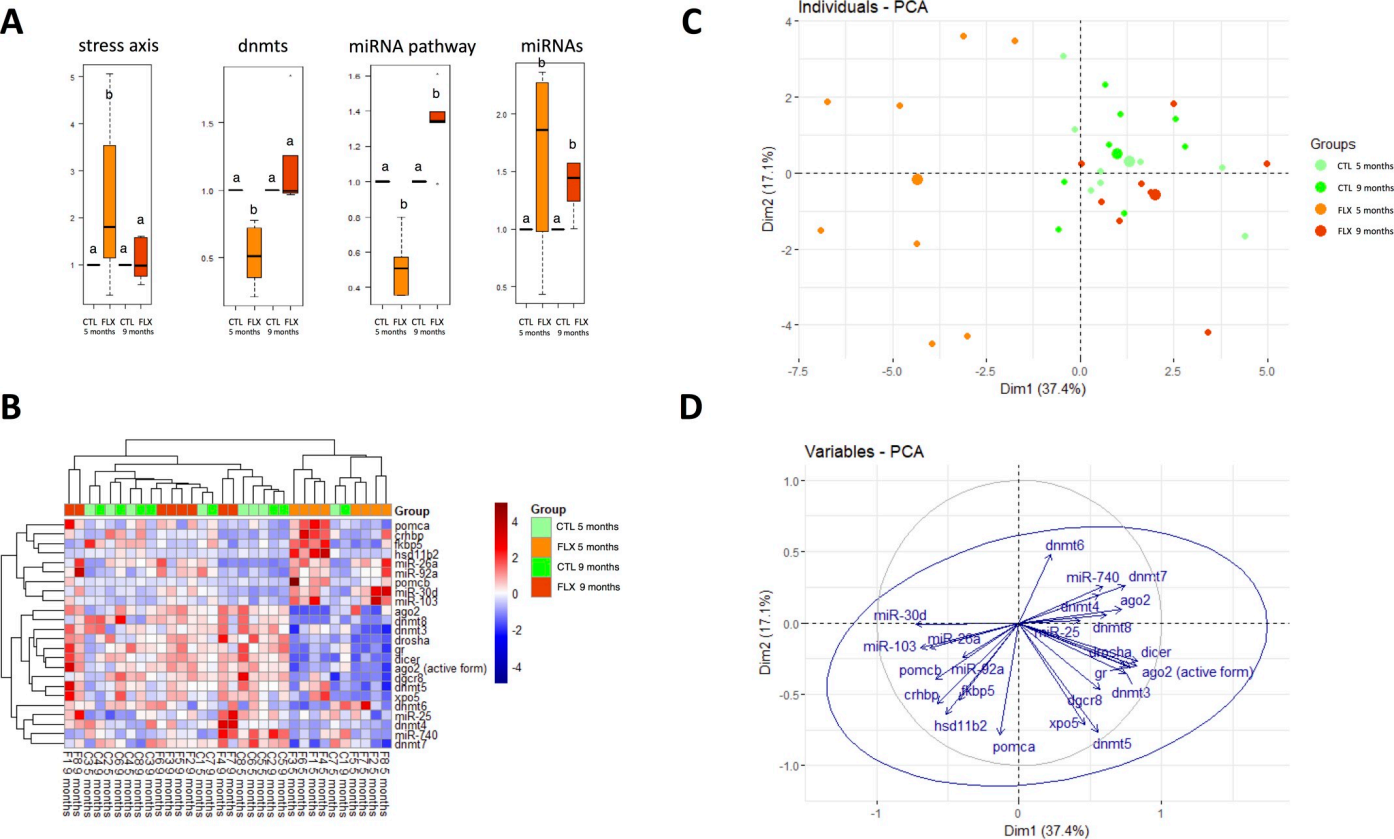
**(A**) Average expression pattern across treatment groups for stress axis transcripts, *de novo* DNA methyltransferases, miRNA biogenesis pathway compounds, and miRNAs. Data are normalized to respective control groups at 5 and 9 months, and expressed as fold-change. Data were analyzed using t-test or Mann-Whitney U test, and significant differences are indicated by different letters within each sampling timepoint. (**B**) Heatmap of the expression of all the transcripts and miRNAs measured, showing the hierarchical clustering of all the egg samples coming from FLX-exposed 5 months old females, together (except for two CTL samples). (**C**) Principal component analysis (PCA) of the samples, using the fold-change expression of each transcript/miRNA as a variable to construct the principal components. The x-axis represents the PC1 while y-axis represents the PC2 (the two main principal components). Together both PC, explain more than fifty percent (54.5%) of the data variability (**D**) Representation of the loadings of all the variables in the PCA performed (**C)**.

In order to address possible involvement of epigenetic mechanisms linked to non-mutational germ-line transmission [60], we profiled *de novo* methyltransferases and components of the miRNA biogenesis pathway. Because eggs are transcriptionally silent, differential *dnmt* profiles do not have immediate effects on maternal transcripts, but may act in active periods of DNA methylation observed following ZGA [60]. Conversely, the miRNA pathway is functionally involved in miRNA-dependent post-transcriptional regulation of maternal transcripts in zebrafish prior to and around ZGA [105–108]. We identified a relatively uniform pattern of *dnmt* paralogue regulation (**Fig 4A–4F; Fig 8A**), consisting of a reduction of *dnmt3*, *dnmt4*, *dnmt7* and *dnmt8* in FLX group eggs compared to CTL at 5 months, suggesting that reduced *de novo* DNA methylation capacity in early larvae may contribute to the maternally inherited reduction of basal cortisol levels in the 12 dpf offspring.

With regard to miRNA pathway genes, we identified a biphasic pattern in several transcripts, with reductions in FLX eggs collected at 5 mpf, and inductions in FLX group eggs at 9 mpf compared to CTL eggs. This pattern suggests a breeding experience and/or age-dependent deposition of miRNA pathway components in eggs (**Fig 8A**). Given the recent identification of functionally different *ago2* splice variants in mouse and frogs [107], we designed two pairs of primers to identify possible splice form-dependent regulation: *ago2* and *ago2* ‘active form’. The *ago2* ‘active form’ is amplified by a primer pair targeting the functional domain spliced-out in mouse and frog oocytes to reduce miRNA regulation of maternal transcripts [107]. While this experimental approach cannot directly determine the presence of splice forms, the identical regulation patterns of *ago2* and *ago2* ‘active form’ suggests that the mechanism above mentioned does either not exist in zebrafish eggs, or is functionally not important in FLX exposed eggs. Nevertheless, the overall signature of miRNA biogenesis components suggests that globally, miRNA impact on maternal transcripts in FLX treated eggs may be reduced in eggs collected at 5 mpf and increased in eggs collected at 9 mpf compared to CTL eggs.

We also profiled specific oocyte miRNA abundance predicted to target stress axis transcripts in order to investigate possible roles for oocyte miRNAs in the observed regulation of maternal stress axis transcripts. In eggs collected at 5mpf, FLX decreased zebrafish-specific *miR-740*, which is predicted to target several stress axis transcripts (**Table 3**). Conversely, *miRNA-26a*, *miRNA-30d*, *miRNA-92a* and *miRNA-103* are all increased in FLX group eggs compared to control eggs collected from F_0_ at 5 mpf. In the case of *miRNA-30d* and *miRNA-92a* transcript abundance remains elevated in FLX group eggs collected at from F_0_ at 9 mpf. Transcript abundance of *miRNA-25* was only increased in FLX group eggs compared to CTL eggs collected from F_0_ at 9 mpf, but not at 5 mpf. To our knowledge, our study is the first to describe contaminant-induced differences in miRNA levels in fish eggs, suggesting that maternal transmission of the reduced cortisol phenotype may be linked to gamete miRNA abundance as has been previously described in rodent models [68]. Based on *in silico* miRNA target prediction, some candidate stress axis genes may be directly targeted. However, clear-cut inverse relationships between miRNA and target transcript abundance suggestive of a principal miRNA-based mode of action in maternal transcript regulation across both timepoints of collected eggs were limited to *mir-740* and *pomcb* and *miR-25* and *fkbp5*. Other predicted relationships were either not or positively correlated, which may be indicative of false positive predictions [109], combinatorial action of unprofiled miRNAs [68], or alternative 3’UTR usage in maternal transcripts [110]. Future studies should therefore utilize transcriptome-level miRNA profiling and morpholino-based injection studies in order to determine mode of action and functional roles of differential miRNA abundance in oocytes in the emergence of the low basal stress phenotype in zebrafish larvae [60].

### 4.3. The egg transcript profile distinguishes eggs derived from FLX exposed females at 5 months from other groups

Regardless of specific functional relationships between measured transcripts, the global transcription profiles of all individual egg batches allowed the distinct clustering of eggs associated with a low basal cortisol phenotype (collected from FLX-F_0_ at 5 mpf), with the exception of two individual eggs collected from CTL-F_0_ at 5 and 9 mpf, respectively (**Fig 8B)**. Using PCA analysis, FLX eggs from FLX-F_0_ collected at 5 mpf separate from other groups along the PC1, which explains slightly more than 1/3 of the overall variability (**Fig 8C**). This axis is loaded mostly with miRNA profiles, while epigenetic profiles and stress axis transcripts also contribute to component PC2 separation and indeed explain some variability observed within FLX eggs collected from F_0_ at 5 mpf itself (**Fig 8D**). This suggests that miRNAs in particular may constitute good candidate molecular markers to link egg quality with reduced stress response to early-life maternal FLX exposure. Of note, miRNAs were also the only measured transcripts exhibiting consistent increases in transcript abundance in FLX-eggs, suggesting possible utility as long-term molecular markers of exposure.

## 5. Conclusions

Our study provides evidence that embryonic exposure to FLX in zebrafish results in maternal transmission of reduced whole body basal cortisol levels in F_1_ offspring larvae. The fact that this effect is dependent on the F_0_ parity and/or life-stage, suggests a sensitive window for maternal transmission. Moreover, we identify a dysregulation in well-characterized stress axis transcripts known to program offspring stress axis in zebrafish as a possible molecular mode of action. Additionally, we describe, for the first time, that developmental exposure to the pharmaceutical FLX can alter not only egg stress axis transcript abundance, but also transcript abundance of epigenetic pathway transcripts. Finally, we show that miRNA abundance in eggs is specifically affected by developmental exposure to FLX, and are indeed an important contributor to separating eggs from developmentally exposed FLX females collected at 5 mpf in clustering and PCA analyses. Finally, we provide evidence that the molecular egg signature allows to cluster eggs that give rise to the reduced basal cortisol phenotype, providing evidence that the time-dependent, maternally transmitted offspring stress axis phenotype may be predicted by the egg molecular profile [60]. Future studies are warranted to functionally investigate the contribution of miRNAs and/or stress axis transcripts to this phenotype, and also to investigate possible longer-term consequences of exposure in F_1_ offspring.

## Supporting information

S1 Data Raw data files. (ZIP)
